# Influence of antidiabetic medications on predictive aptitude of TyG index for HFpEF

**DOI:** 10.1002/clc.23968

**Published:** 2023-01-01

**Authors:** Tomonari Shimoda, Ryusuke Imanishi, Noriaki Kou, Yui Okamura, Hiroshi Ito, Nobutake Shimojo

**Affiliations:** ^1^ School of Medicine and Health Sciences, College of Medicine University of Tsukuba Tsukuba Japan; ^2^ Department of Internal Medicine, Division of General Internal Medicine Tokyo Medical University Ibaraki Medical Center Inashiki‐gun Ibaraki Japan; ^3^ Division of Hospital Medicine University of Tsukuba Hospital Tsukuba Japan

To the Editor,

Liao et al.^1^ successfully demonstrated the ability of triglyceride glucose (TyG) index in the prediction of heart failure with preserved ejection fraction (HFpEF) in patients with essential hypertension. However, the authors did not consider the potential confounding effects of therapeutics for dyslipidemia and diabetes mellitus on the predictive aptitude of TyG index. TyG index is an indicator calculated using triglyceride and fasting blood glucose levels. Thus, lipid and glucose‐lowering drugs may influence the value of TyG index. Indeed, a contemporary study revealed that SGLT2 inhibitors influence the result of TyG index.^2^ Furthermore, recent studies suggest the positive effect of sodium‐glucose transporter 2 inhibitors on the outcomes in HFpEF patients. Future validation studies are needed to determine the usefulness of TyG index.

## AUTHOR CONTRIBUTIONS

All authors verify their contribution to the production and writing of this manuscript.

## CONFLICT OF INTEREST

The authors declare no conflict of interest.
